# Supplementary material for: CPPF, A Novel Microtubule Targeting Anticancer Agent, Inhibits the Growth of a Wide Variety of Cancers
Source: Int J Mol Sci. 2020 Jul 7;21(13):4800. doi: 10.3390/ijms21134800 (PMC7370279; doi:10.3390/ijms21134800)
Supplement: Supplementary file 1 [file ijms-21-04800-s001.zip › CPPF Supplemental informatics-V11.docx]

**CPPF, A Novel Microtubule Targeting Anticancer Agent, Inhibits the Growth of a Wide Variety of Cancers.**

Ho Jin Han^1, 2, §^, Chanmi Park^1, §^, Joonsung Hwang^1, §^, Thimmegowda N.R.^1, #^, Sun-Ok Kim^1^, Junyeol Han^1, 2^, Minsik Woo^1, 3^, Shwetha B^1, Ŧ^, In-Ja Ryoo^1^, Kyung Ho Lee^1^, Hyunjoo Cha-Molstad^1^, Yong Tae Kwon^4,^ *, Bo Yeon Kim^1, 2,^ *, and Nak-Kyun Soung^1,^ *

^1^ Anticancer Agent Research Center, Korea Research Institute of Bioscience and Biotechnology, Ochang, Cheongju, 28116, Rep. of Korea

^2^ Department of Biomolecular Science, University of Science and Technology, Daejeon, 34113, Rep. of Korea

^3^ College of Pharmacy and Medical Research Center, Chungbuk National University, Osong, Cheongju, 28160, Rep. of Korea

^4^ Protein Metabolism Medical Research Center and Department of Biomedical Sciences, College of Medicine, Seoul National University, Seoul, 03080, Rep. of Korea

^§^ These authors equally contributed to this work.

Current address:

^#^Government S.K.S.J.T Institute; Bangalore, Karnataka, 560001, India

^Ŧ^Visvesvaraya Technological Institute, CPGS, Muddenahalli 562101, India

^*^Correspondence:Yong Tae Kwon (yok5@snu.ac.kr; Tel.:+82-2-740-8547; Fax: +82-2-3673-2167), Bo Yeon Kim (bykim@kribb.re.kr; Tel.: +82-43-240-6163; Fax: +82-43-240-6259), Nak-Kyun Soung (soungnak@kribb.re.kr; Tel.: +82-43-240-6165; Fax: +82-43-240-6259)

**Contents:**

- ***Synthetic procedure, ESIMS, ^1^H NMR & ^13^C NMR Spectrum of compound CPPF***
- ***Xenograft Mouse Model***

**Supplemental Materials and Methods**

***1. Synthesis of 5-(3-chlorophenyl)-N-(pyridin-3-yl)furan-2-carboxamide (CPPF)***

**Experimental section:**

Unless otherwise noted, all reactions were run under a nitrogen (N_2_) atmosphere in oven dried glassware. Reactions were stirred using teflon-coated magnetic stirrer bars. Reactions were monitored using thin layer silica gel chromatography (TLC) using silica gel (Kieselgel 60 F254) plates with fluorescent indicator from Merck. Plates were visualized by treatment with UV, acidic *p*-anisaldehyde, phosphomolybdic acid and 10% ethanolic H_2_SO_4_ stain, with gentle heating. The solvents were removed under reduced pressure using standard rotary evaporators. Products were purified via silica gel (particle size 40-63 µm, Merck) flash column chromatography using the solvent systems indicated.

All of the solvents and reagents used were obtained commercially and used as such unless noted otherwise. Dichloromethane (CH_2_Cl_2_) solvent was freshly distilled over calcium hydride (CaH_2_) under nitrogen. Anhydrous dimethylformamide (DMF) were obtained from Sigma–Aldrich. Ethyl acetate (EtOAc), hexanes were obtained from Duksan pure chemicals, diethylether and toluene were obtained from Junsei chemicals and ethanol was obtained from Burdik and Jackson. 3-Chlorophenylboronic acid, 5-Bromo-2-furoic acid, Cesium carbonate (Cs_2_CO_3_), Tetrakis(triphenylphosphine)palladium(0) (Pd(PPh_3_)_4_), 3-Aminopyridine, *N*-(3-Dimethylaminopropyl)-*N*′-ethylcarbodiimide hydrochloride (EDCI),1-Hydroxybenzotriazole hydrate (HOBt) and 2.0M Hydrogen chloride solution in diethyl ether were purchased from Sigma–Aldrich.

NMR spectra were measured on a Varian INOVA 400 (^1^H at 400 MHz, ^13^C at 100 MHz) magnetic resonance spectrometer, as noted. ^1^H chemical shifts are reported relative to the residual solvent peak (DMSO = 2.5 ppm) as follows: chemical shift (δ), multiplicity (s = singlet, d = doublet, dd = dublet of dublet, t = triplet, q = quartet, m =multiplet), integration, coupling constant(s) in Hz. ^13^C chemical shifts are reported relative to the residual deuterated solvent ^13^C signals (DMSO = 39.5 ppm). ESIMS analyses were performed on a Finnigan MAT,Navigator.

**Procedure for the synthesis of intermediate compound 5-(3-chlorophenyl)furan-2-carboxylic acid (3)**

3-Chlorophenylboronicacid **1** (5 g, 31.97 mmol), 5-Bromo-2-furoic acid **2** (7.32 g, 38.4 mmol) and cesium carbonate (Cs_2_CO_3_) (41.66 g, 128 mmol) were dissolved in toluene (100 mL) and ethanol (100 mL) under nitrogen atmosphere was added tetrakis(triphenylphosphine)palladium (0) (Pd(PPh_3_)_4_) (0.369 g, 0.319 mmol) and the resulting reaction mixture was heated to 85 ^o^C and stirred for 3 h. The solvents were removed under vacuo. The resulting mixture was diluted with H_2_O and ethyl acetate. The layers were separated, and the aqueous layer was acidified with dilute HCl and extracted with ethyl acetate (3 x 200 mL). The combined organic layers were dried over anhydrous Na_2_SO_4_, filtered, and concentrated in vacuo. The resulting intermediate compound was purified by recrystallization from diethyl ether and hexane to yield 5-(3-chlorophenyl)furan-2-carboxylic acid (**3**) (3.9 g, 55% yield) as a pale green solid.

**ESIMS** found: *m*/*z* 221.2 [M-H]^-^.

**Procedure for the synthesis of 5-(3-chlorophenyl)-N-(pyridin-3-yl)furan-2-carboxamide hydrochloride (CPPF)**

A solution of 5-(3-chlorophenyl)furan-2-carboxylic acid **3** (2g, 8.98 mmol) in anhydrous dimethylformamide (10 mL) was cooled to 0 ^o^C in an ice bath. Triethylamine (TEA) (1.25 mL, 26.9 mmol) and *N*-(3-Dimethylaminopropyl)-*N*′-ethylcarbodiimide hydrochloride (EDCI) (2.58 g, 13.4 mmol) were added. The mixture was stirred at 0 ^o^C for 30 min and then 1-Hydroxybenzotriazole hydrate (HOBt) (1.43 g, 9.88 mmol) and 3-Aminopyridine **4** (1.01 g, 10.7 mmol) were added. The resulting reaction mixture was stirred at 0 ^o^C for 30 min and then warmed to room temperature and stirred for 6 h.

The reaction mixture was diluted with H_2_O and ethyl acetate. The layers were separated, and the aqueous layer was extracted with ethyl acetate (3 x 200 mL). The combined organic layers were dried over anhydrous Na_2_SO_4_, filtered, and concentrated in vacuo. The resulting residue was purified by silica gel flash column chromatography using hexane: ethyl acetate (1:1) mobile phase to yield the compound 5**-**(3-chlorophenyl)-N-(pyridin-3-yl)furan-2-carboxamide (2.2 g) as a yellow solid. **R*_f_* =** 0.35 (hexane: ethyl acetate = 1:2).

The pure compound 5-(3-chlorophenyl)-N-(pyridin-3-yl)furan-2-carboxamide (2.2 g) was dissolved in dichloromethane (20 mL), cooled to 0 ^o^C and was then added 2 M hydrogen chloride solution in diethyl ether (10 mL). The resulting mixture was stirred at 0 ^o^C for 30 min and then at room temperature for 4h. The precipitate thus separated out was filtered and dried under vacuo. 2.1 g (70 %) of 5-(3-chlorophenyl)-N-(pyridin-3-yl)furan-2-carboxamide hydrochloride (**CPPF**) obtained as white crystalline solid.

**^1^H NMR** (400 MHz, DMSO-*d_6_***)** δ ppm**:** 11.424 (1H, s, O=C-NH-), 9.413-9.408 (1H, d, Ar-H, *J* = 2 Hz), 8.906-8.885 (1H, d, Ar-H, *J* = 8.4 Hz), 8.655-8.642 (1H, d, Ar-H, *J* = 5.2 Hz), 8.103-8.101 (1H, d, Ar-H, *J* = 0.8 Hz), 8.038-8.002 (1H, dd, Ar-H, *J* = 8.44 Hz, 5 Hz), 7.976-7.955 (1H, dd, Ar-H, *J* = 7.7, 0.9 Hz), 7.740-7.730 (1H, d, Ar-H, *J* = 4 Hz),7.552-7.512 (1H, dd, Ar-H, *J* = 7.9, 7.9 Hz), 7.475-7.456 (1H, d, Ar-H, *J* = 7.9), 7.371-7.364 (1H, dd, Ar-H, *J* = 3.54, 0.9 Hz); **^13^C NMR**(100 MHz, DMSO-*d_6_*): δ ppm**:**109.561, 118.990, 123.323, 124.208, 126.947, 128.731, 130.924, 133.790, 133.932, 134.810, 137.191, 138.051, 145.844, 154.479, 156.427; **ESIMS** found: *m*/*z* 333.3 [M-H]^-^,335.2 [M-H+2]^-^.

**Mass spectrum of intermediate compound 3**


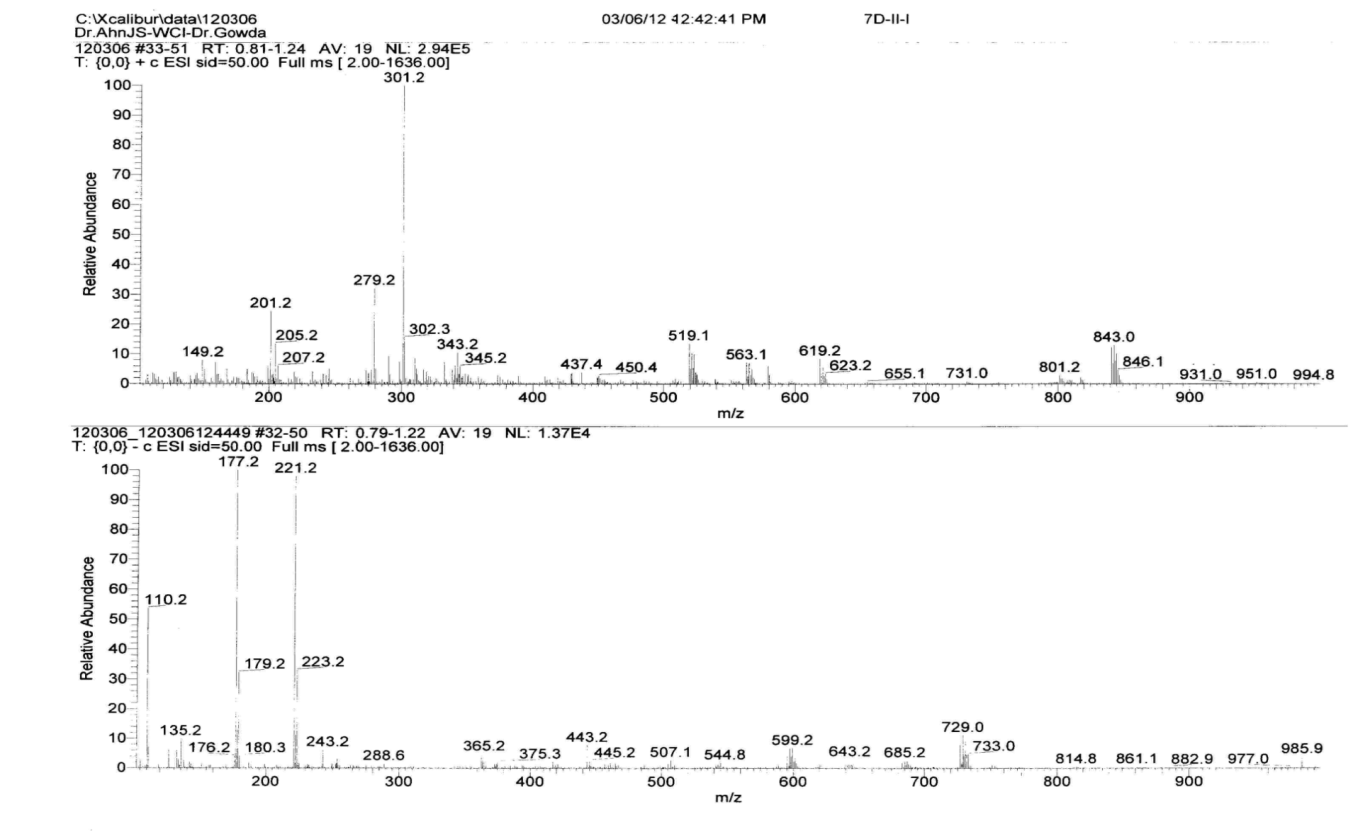


Compound-3

**Mass spectrum of compound-CPPF**

^
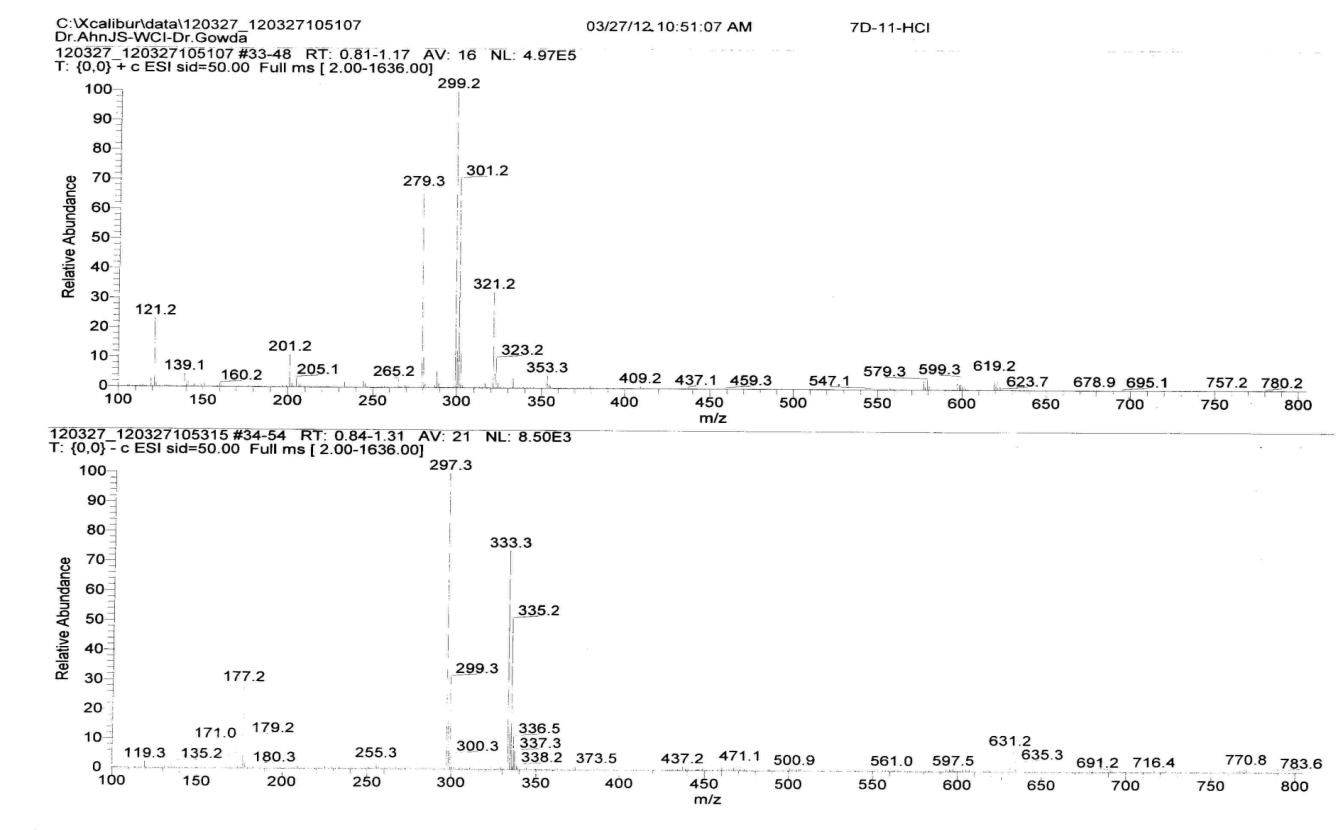
^

**^1^H NMR spectrum of compound-CPPF**


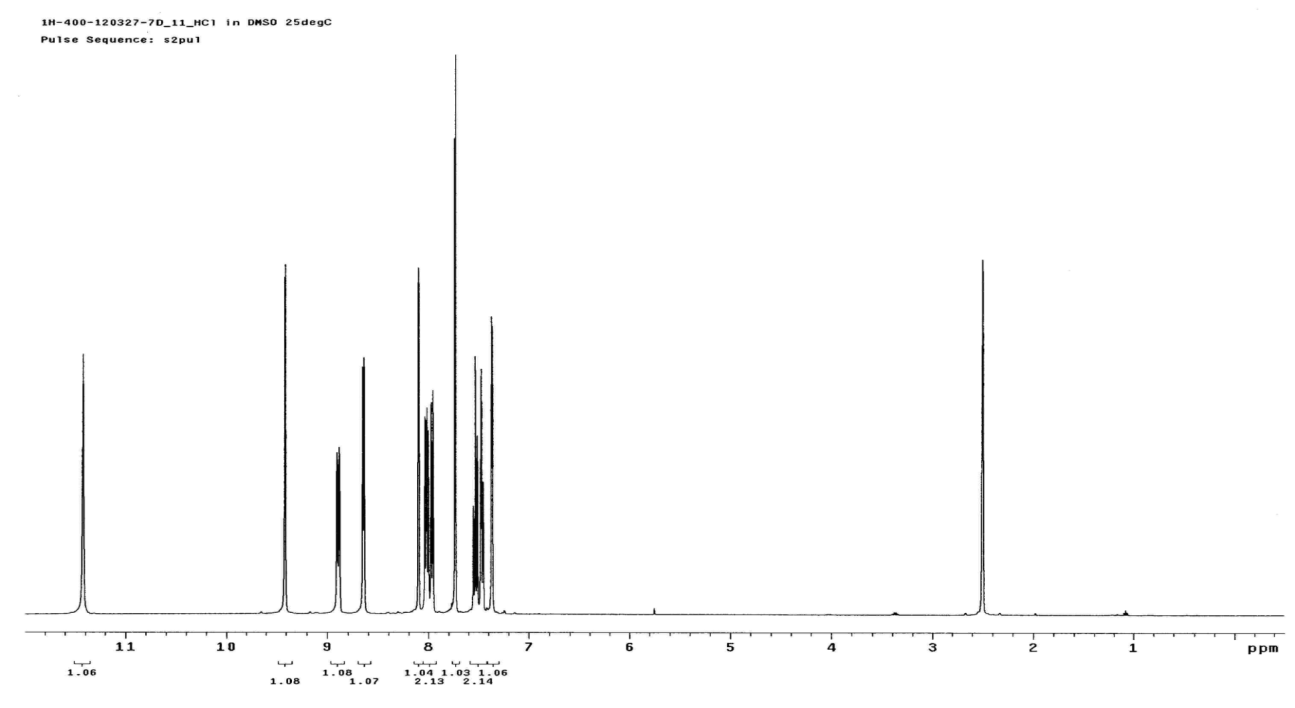


**^13^C NMR spectrum of compound-CPPF**


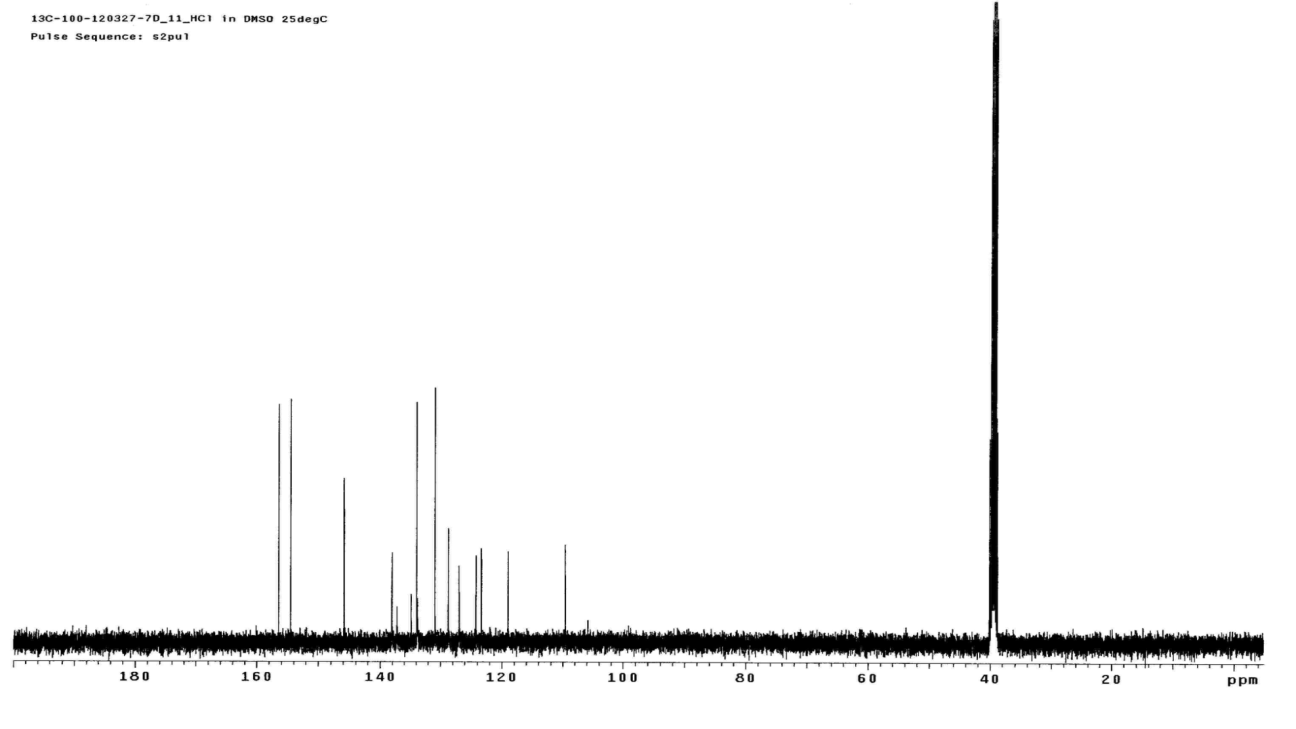


**2. *Xenograft Mouse Model***

The male Balb/C nude mice (6W) were purchased from the Daehan biolink. 3X10^5^ HeLa cells/mouse were inoculated subcutaneously into mice. When tumor volumes reached 100mm^3,^ the mice were divided into 2 groups. Each group consisted of 6 mice, and 3 times a week, IP injections of 50 mg/kg CPPF or vehicle only were done until tumor volume reached 1000mm^3^. Then, the final volume were measured.

**Supplementary Figure legends**

**Supplemental Figure 1**

**Antitumor effect of CPPF in a xenograft model.**

1. HeLa cells were subcutaneously injected in mice. When tumors reached an average size of 100mm^3^, the vehicle only or vehicle plus 50mg/kg of CPPF was injected 3 times a week. Images were taken at the end of the experiment. **B**. The final tumor volumes were measured .Data are presented mean and each mice.
