# Supplementary material for: CPPF, A Novel Microtubule Targeting Anticancer Agent, Inhibits the Growth of a Wide Variety of Cancers
Source: Int J Mol Sci. 2020 Jul 7;21(13):4800. doi: 10.3390/ijms21134800 (PMC7370279; doi:10.3390/ijms21134800)
Supplement: Supplementary file 1 [file ijms-21-04800-s001.zip › Supplemental table-V11.docx]

**Supplemental Table. Diverse organ origin cancer cell line CPPF IC50s**

| **Origin Organ** | **Cell name** | **IC50(µM)** |
| --- | --- | --- |
| Cervical | HeLa | 0.692±0.018 |
| Leukemia | Jurkat | 0.222±0.001 |
|  | WehI3 | 0.241±0.014 |
|  | U937 | 0.921±0.054 |
|  | P388 | 1.372±0.175 |
|  | EL4 | 1.123±0.215 |
| Hepatoma | HepG2 | 2.826±0.56 |
|  | Hep3B | 1.274±0.019 |
| Stomach | SNU484 | 0.673±0.006 |
|  | SNU601 | 1.812±0.108 |
| Lung | NCI-H1299 | 0.755±0.005 |
|  | A549 | 3.151±0.288 |
| Prostate | PC3 | 4.063±0.023 |
| Skin | A431 | 1.235±0.132 |

Human cervical cancer (HeLa), Leukemia (Jurkat, WheI3, U937, P388, EL4), Hepatoma (HepG2, Hep3B), Stomach (SNU484, SNU601), Lung (NCI-H1299, A549), Prostate (PC3) and Skin (A431) were treated with 0 to 10 µM CPPF for 4 days. Cell viability was measured and calculated in an MTT assay. Data presents IC_50_ (µM) by Mean ± Standard deviation of three independent experiments.
