# Supplementary figures and images for: CPPF, A Novel Microtubule Targeting Anticancer Agent, Inhibits the Growth of a Wide Variety of Cancers
Source: Int J Mol Sci. 2020 Jul 7;21(13):4800. doi: 10.3390/ijms21134800 (PMC7370279; doi:10.3390/ijms21134800)

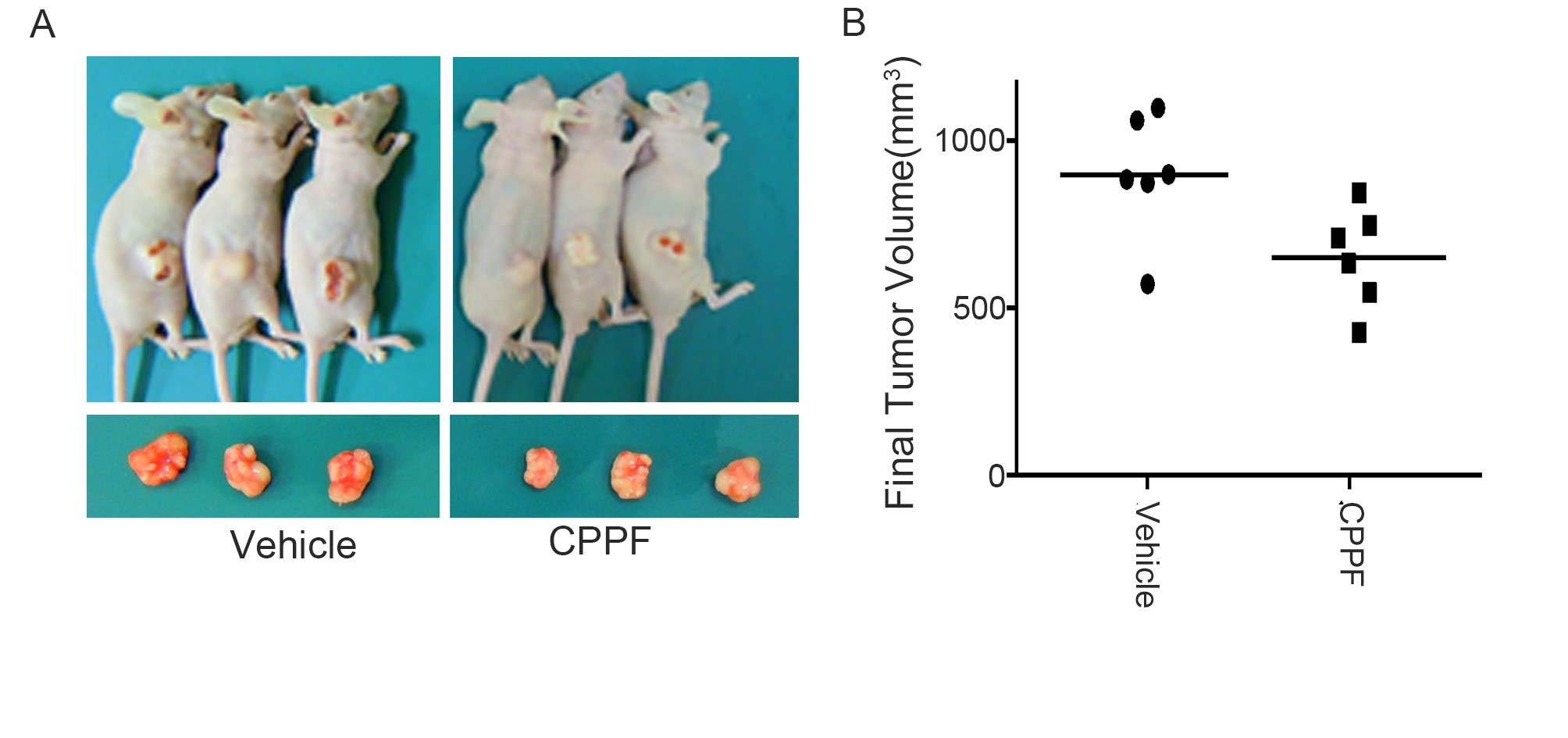

Supplement: Supplementary file 1 [file ijms-21-04800-s001.zip › Supplemental Figure 1-V11.jpg]
